# The Expensive-Tissue Hypothesis in Vertebrates: Gut Microbiota Effect, a Review

**DOI:** 10.3390/ijms19061792

**Published:** 2018-06-17

**Authors:** Chun Hua Huang, Xin Yu, Wen Bo Liao

**Affiliations:** 1 Key Laboratory of Southwest China Wildlife Resources Conservation (Ministry of Education), China West Normal University, Nanchong 637009, Sichuan, China; 18380586112@163.com (C.H.H.); 15183557383@163.com (X.Y.); 2 Institute of Eco-adaptation in Amphibians and Reptiles, China West Normal University, Nanchong 637009, Sichuan, China

**Keywords:** gut microbiota, diet, gut size, brain size, the Expensive-Tissue Hypothesis

## Abstract

The gut microbiota is integral to an organism’s digestive structure and has been shown to play an important role in producing substrates for gluconeogenesis and energy production, vasodilator, and gut motility. Numerous studies have demonstrated that variation in diet types is associated with the abundance and diversity of the gut microbiota, a relationship that plays a significant role in nutrient absorption and affects gut size. The Expensive-Tissue Hypothesis states (ETH) that the metabolic requirement of relatively large brains is offset by a corresponding reduction of the other tissues, such as gut size. However, how the trade-off between gut size and brain size in vertebrates is associated with the gut microbiota through metabolic requirements still remains unexplored. Here, we review research relating to and discuss the potential influence of gut microbiota on the ETH.

## 1. Introduction

Vertebrates have coevolved with a diverse range of symbiotic gut bacteria and other microorganisms that populate the intestinal tract, known collectively as the gut microbiota [1,2]. Resident microbiota exhibit a mutually beneficial relationship with their host. In particular, products of the microbes can contribute to the evolutionary fitness of the host [3]. The diversity and composition of the gut microbiota among individuals of a host species often varies topographically and temporally [4,5], with particular bacterial species being associated with the host’s food intake [6] and with consequences for nutrient utilization and energy metabolism [7].

Subsequent shaping of the microbial landscape is then driven by a series of complex and dynamic interactions throughout life, including diet [5]. On this issue, previous studies have reported that dietary type is a dominant force in determining the composition and diversity of an individual’s gut microbiota [7,8,9,10,11,12]. For instance, a high-fat diet increases the level of Bacteroidete and decreases the level of Firmicute in children’s guts [8]. It has also been shown by several studies that diet quality is highly associated with gut size. Specifically, a higher-quality diet results in smaller gut size in grouse, fish, and primates [13,14,15,16,17,18].

There are interactions between intestinal bacteria and their host digestive system [19]. The microbiota takes part in shaping the development, physiology, and morphogenesis (e.g., blood vessel density and gut size) of the digestive system [20,21,22,23], and in turn, the composition of the microbiota can be shaped by the digestive system as well. This communication between the microbes and the digestive system is passed via direct chemical substrate and/or signaling processes that affect tissues such as the gut, liver, and brain [5,20,21,22,23]. Through complex interactions, metabolic reactions can be sequentially modulated by multiple bacterial genomes, so that the microbiome and host work in combination for the metabolism of substrates. An example result of these interactions are short-chain fatty acids (SCFAs), metabolism products [5] that play an important role in the host’s food intake [6,24], gut nutrient utilization, energy metabolism, and development [21,25,26,27,28,29].

The developmental trajectory of the microbiome can modulate the metabolic phenotype of the host and hence greatly influence the host’s biochemistry [5,21,30]. Several previous studies have shown that gut size can be affected by food quality through changes to the energy harvested and nutrition absorbed [13,14,15,17]. Further, studies have shown that cecal size and intestinal morphology can be influenced by the intestinal microbiota [20,21,22,23]. Gut microbiota is likely to increase the ratio of nutrients absorbed to food intake through the digestion of complex carbohydrates in food which the host is unable to digest. This increase then results in an increase of net energy input to the host [25,31,32,33,34,35]. The theory that microbiota mediates the interactions between diet and gut size can then be deduced from this relationship.

Interestingly, it has been found that gut size has seen coevolution with brain size [15,36,37,38,39,40,41,42]. Both the brain and gut are energetically costly organs in the vertebrate body [15]. The Expensive-Tissue Hypothesis (ETH) states that the high energetic expenditure of larger brains requires a matching decrease in other energetic-consuming organisms (e.g., guts) [15,36,37,38,39,41,42]. However, studies have refuted the ETH [43,44,45,46,47] and proposed the trade-off hypothesis, which suggests that instead of tissue investment, a series of trade-offs with other energetically costly traits such as body maintenance, locomotion, development, and reproductive investment (e.g., testes mass) can compensate for the cost of increased brain size [39,43,44,45,46,47,48,49,50,51]. Nonetheless, the gut plays an important role in brain size evolution and can be viewed as one of these energy trade-offs. For instance, larger-brained individuals have evolved smaller guts in several taxa [36,40,41]. For example, there is a negative correlation between brain size and gut size in guppies (Poecilia reticulata) [41] and the Omei Wood Frog (Rana omeimontis) [40].

The trade-off between gut and brain size can be mediated by meeting either indirect chemical requirements or direct energy metabolism demands through alternative methods [15]. Intestinal chemical requirements may be achieved by gut microbial products as well as intestinal digestive absorption products [52]. For example, microbiota can provide long-chain fatty acids for the brain [26,29,33] and short-chain fatty acids (butyrates) for the colonic epithelium [5,25,33]. For direct energy, the microbial inhabitants of the gut have been shown to affect metabolic processes (e.g., energy extraction from food) [33], which require huge amounts of energy substrates produced by these bacteria and oxygen [15,53]. Furthermore, SCFAs acted as an important source of energy for colonocytes and as a substrate for gluconeogenesis, and are thus important for modulating the central metabolism [7].

Here, we review how gut microbiota responds to diet quality and how it influences host metabolism by improving energy yield from food and modulating dietary or host-derived compounds that alter host metabolic pathways. We then discuss how these processes mediate the trade-off between gut size and brain size and how they support the ETH.

## 2. Influences of Gut Microbiota on Host Digestive System

Major organs of vertebrate hosts, such as the skin and gut, are populated by trillions of microbial residents [54,55]. Among host organs, the large intestine is the main place for gut microbiota to inhabit. The dominant microflora mainly consists of Firmicutes, Bacteroidetes, Actinobacteria, Proteobacteria, and Fusobacteria [56,57].

The gut microbiota of infants is seeded at birth and shaped by diverse delivery modes [5,19]. Research measuring the microbial community structure of maternal and fetal samples collected from healthy mothers and infants has shown that, at the phylum level, the meconium microbe structure is similar to the structures of the maternal amniotic fluid, vaginal fluid, and breast milk [19]. Additionally, the microbiota of babies delivered vaginally is dominated by Lactobacillus, Prevotella, and Atopobium, whereas babies delivered by Cesarian section have microbiota that more closely resembles that of the maternal skin, with staphylococci being a dominant early member [5,19]. Colonization of the infant gastrointestinal tract is realized via contact with maternal amniotic fluid and microbiota in utero in which the mother’s vaginal and fecal microbiota inoculate the baby [19,58]. These transferred microbiota consist of facultative anaerobes that can in turn create anaerobic conditions, promoting the development of obligate anaerobes [59].

The intestine microflora, considered a postnatally acquired organ, perform different functions on the systems of the host [19,35,57,60,61,62]. The host’s digestive system has particularly been influenced [60], as the capability of the microbiota to contribute substrates and energy to the host via several symbiotic mechanisms is maximized in the gut. For example, numerous enzymes required to de-modify, liberate, transport, and metabolize component monosaccharides are not encoded within the human genome [7,63]. Instead, genes of glycosidase and lyase are contained by microbial species such as Bacteroides thetaiotaomicron and B. ovatus and hence those species are able to utilize most of the main vegetal food residue by employing the host’s glycans (e.g., mucus-associated glycoproteins) when they arrive at the caecum, colon, rumen, and hind gut [25,31,32,33,34,35]. Another example of this relationship is the degradation of resistant starch particles that arrive in the human colon are by Ruminococcus bromii of the Firmicutes family. Finally, Bateroids spp. are another example of symbiotic microbiota. These utilize a diverse variety of host-derived glycans [35] before fermenting oligosaccharides and monosaccharides to yield short-chain fatty acids (e.g., acetate, propionate, and butyrate) and gases (e.g., hydrogen, carbon dioxide, methane, and hydrogen salphide) as end products [5,25,33,58].

**Figure 1:**
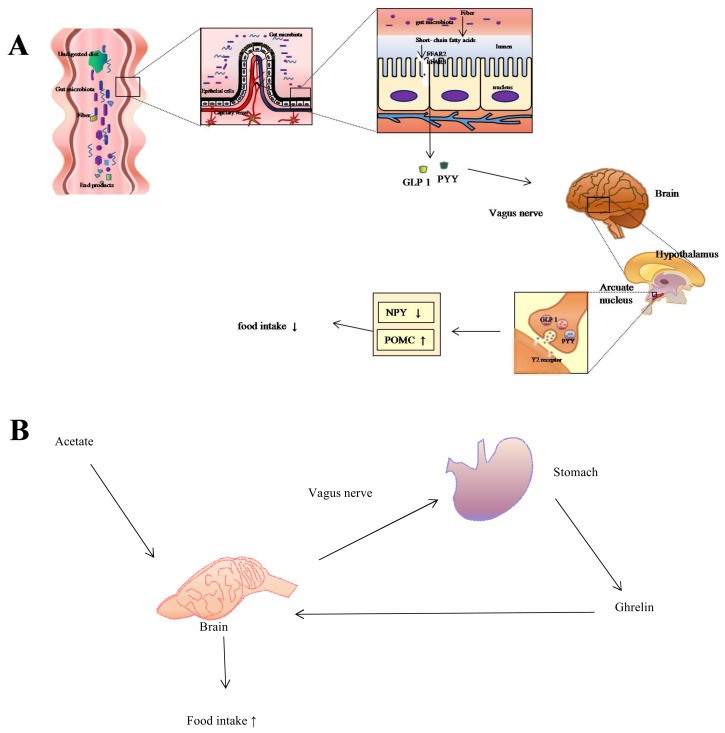
Regulation of food intake via gut-brain pathway. (**A**) When undigested dietary residue arrives at the caecum, colon, rumen, and hind gut, the bacteria populated in the intestines ferment them to short-chain fatty acids (SCFAs) (e.g., acetate, propionate, and butyrate). Then free fatty acid receptor 2 (FFAR2) and free fatty acid receptor 3 (FFAR3) on the L-cells interact with SCFAs to trigger the secretion of anorectic hormones, including peptide YY (PYY) and glucagon-like peptide 1 (GLP1). PYY and GLP1 then preferentially bind to the Y2 receptor located on the arcuate nucleus of the hypothalamus via the vagus nerve, which further increases the expression of the anorexigenic pro-opiomelanocortin (POMC) neuropeptide while decreasing the expression of neuropeptide Y (NPY), thus managing to control food intake; (**B**) Microbiota ferment dietary nutrients, digesting them to SCFAs. Acetate molecules then stimulate the vagus nerve, which triggers the stomach to secrete the “hunger hormone” ghrelin, leading to the increase of food intake.

**Figure 2:**
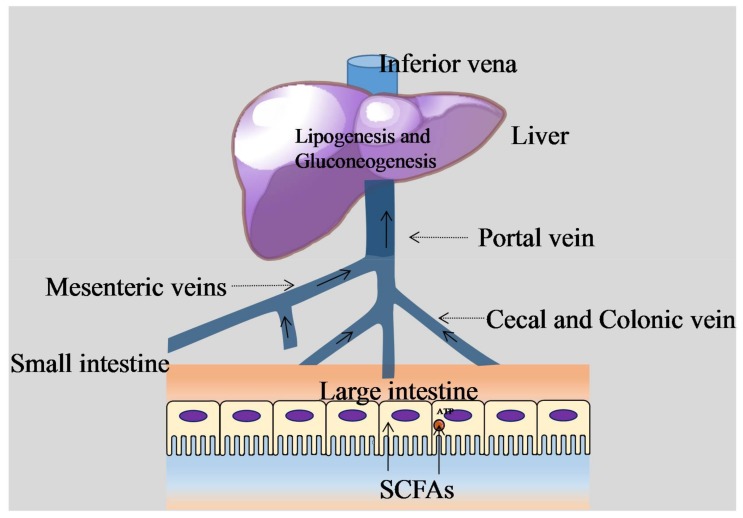
SCFAs in liver circulation and central metabolism. Butyrate, propionate, and acetate enter the liver via the portal vein after passing through the mesenteric veins, cecal veins, and colonic veins. They are then utilized for lipogenesis and gluconeogenesis in the liver, the products of which then enter blood circulation. SCFAs are an important source of energy (in the form of ATP) for colonocytes and for gluconeogenesis in the modulation of the central metabolism.

SCFAs play an important role in nutrition and physiology [25,26,29] and are attributed to various species of Clostridial clusters IV and XIV a of Firmicutes [7] as well as those phylum of bacteria with putative genes coding for cellolose, β-glucosidase, and xylan 1,4-β-xylosidase [64]. When acting as a signal, SCFAs are related to food intake (Figure 1). On the one hand, free fatty acid receptor 2 (FFAR2) and free fatty acid receptor 3 (FFAR3) take part in controlling anorectic hormones, including peptide YY (PYY) and glucagon-like peptide 1 (GLP1), which exist to reduce food intake [65]. On the other, the ‘hunger hormone’ ghrelin from the stomach takes part in increasing the amount of food intake, which is triggered by signals from the vagus nerve in response to acetate molecules [24]. When serving as the substrate for gluconeogenesis and energy production, short-chain fatty acids provide energy for colonic epithelial cells through oxidation [24,66] (Figure 2). For instance, germ-free rodents have reduced intestinal levels of SCFAs [67] and thus reduced energy harvest, requiring an increase food intake in compensation [68]. SCFAs are also involved in energy production in the liver, where butyrate and propionate are substrates for gluconeogenesis [26], and acetate synthesizes long-chain fatty acids and glutamic acids [26,29,33]. Further, as a type of vasodilator that is capable of affecting intestinal angiogenesis, SCFAs improve microcirculation to increase absorption and utilization of nutrition in the colon and distal ileum [23,27,29,33,69,70].

SCFAs are also associated with intestinal transit [27,71] through the stimulation of gastrointestinal secretions of gastrin and motilin. When gastric acid secretion is increased, gastric motility and gastrointestinal mucosal cell growth are also increased. Motilin is involved with the physiological regulation of gastric motility and stimulates the secretion of pepsin and pancreatic juice [29]. These respective effects enhance digestion. Another effect of SCFAs comes in the form of butyrate acting as a histone deacetylase (HDAC) inhibitor which alters the proliferation, differentiation, and modulation of gene expression in mammalian colonic epithelial cells [21,23,26,72]. Specifically, butyrate-responsive promoters regulate butyrate at first, then butyrate recruits HDAC1 and HDAC2 under the mediation of specificity proteins 1/specificity proteins 3 (Sp1/Sp3) at their respective binding sites. The HDAC2 is then phosphorylated by protein kinase CK2 (formerly known as casein kinase II). Histone hyperacetylation and transcriptional activation of the p21Waf1/Cip1 gene are caused by the inhibition of Sp1/Sp3 which is related to HDAC activity; Cyclin-dependent kinase 2 (CDK2) activity is inhibited by p21Waf1/Cip1 and which serves as a method to arrest cell cycling. Depending on the nonproliferating cell’s background, they will differentiate through various apoptotic pathways [72].

The composition of the intestine microbiota can affect the type and the amount of SCFAs available to the host. For instance, *Eubacterium rectale* and Firmicutes *F. prausnitzii* produce butyrate; lactate can be converted into butyrate by *Eubacterium hallii* and *Anaerostipes* spp.; Bacteroidetes and Veillonellaceae (Firmicutes) produce propionate, a type of hexose sugar through the succinate or acrylate pathway [73]; acetogenic bacteria such as *Blautia hydrogenotrophica* (Firmicutes) produce acetate. The above-mentioned instances indicate that certain bacteria might represent specialists whose primary function is to produce specific products. Moreover, interactions between bacteria species within the gut affect their utilization of polysaccharides and the products they output [33,74]. In order to investigate contributions of Archaea to digestive health, Samuel and Gordon (2006) colonized germ-free mice with *Bacteroides thetaiotaomicron*, both with and without *M. smithii* or the sulfate-reducing bacterium *Desulfovibrio piger*. Whole genome transcriptional profiling and mass spectrometry of *B. thetaiotaomicron* revealed that unlike *D. piger*, *M. smithii* directs *B. thetaiotaomicron* to focus on the fermentation of dietary fructans to acetate. *M. smithii* in turn uses *B. thetaiotaomicron*-derived formate for methanogenesis. *B. thetaiotaomicron*-*M. smithii* cocolonization produced a significant increase in host adiposity compared with mono-associated, or *B. thetaiotaomicron*-*D. piger* bi-associated, animals [74]. In this manner the interaction network of gut microbiota can result in more efficient carbohydrate fermentation and increases energy absorption in the gut.

By guaranteeing a conversion in the relative production of diverse fermentation products, gaseous compounds produced in the colon can be influenced by Hydrogen disposal, which is also capable of impacting the metabolism of hydrogen-producing fermentative bacteria [75,76]. Specifically, methanogenesis results in slower transit time and likely reflects the slow development of methanogenic Archaea, which utilize hydrogen and carbon dioxide to produce methane [77]. This has been found to prolong intestinal transit [78], which can increase the turnover time of digestion and in turn increase the efficiency of nutrient utilization [79].

It is likely that alterations in the production of the major SCFAs have an important influence on physiology. Changes of SCFA production are associated with gut microbiota, and the modulation and fermentation capacity of the microbiota may affect the absorption, energy harvest, and growth of the digestive system [33], which might be crucial when individuals face food scarcity [58].

## 3. Gut Microbiota Associated with Diet Affects Gut Size

The gut microbiota of an infant changes substantially from weaning to attaining a normal diet and then remains stable throughout adulthood [58,80,81,82]. Its composition is shaped by a variety of factors, including host traits (e.g., metabolism and gut condition) and environmental factors (e.g., diet) [19,56,57]. In turn, the gut microflora has multiple essential functions that it is capable of performing on a host’s digestive tract (e.g., development and morphogenesis).

Distinct differences exist in gut environment for different anatomical areas in terms of digestion flow rates, host secretions, and oxygen tension [58]. For example, in terms of the pH state, the development of Bacteroides spp. gets curtailed when pH values < 6 at short-chain fatty acid concentrations (50–100 nM), while many Firmicutes can adapt to acidic pH well [83]. With regard to oxygen gradients, Bacteroides spp. possesses a cytochrome bd oxidase [84,85,86] that allows for its development in environments of concentrated nanomolar oxygen [87]. Most Firmicutes, on the other hand, are considered to be strict anaerobes [88]. The large intestine, which features the largest microbial community, provides nearly neutral pH and harbors low oxygen concentration. Conversely, microbiota in the small intestine face difficult conditions, as it features a short transit time (3–5 h), a high concentration of bile, and low pH [89,90], leading to the existence of less microbiota.

Polysaccharides serve as the primary and easiest avenue for microbiota to modulate the host’s metabolism [8,9,11]. As non-digestible carbohydrates are important sources of energy for several members of the colonic microbiota, host diet (both long-term and short-term) influences bacterial diversity [8,11]. This bacterial diversity increases as mammals shift from carnivorous to omnivorous and finally to herbivorous diets [91]. For example, research has shown that children who come from a rural African village where the diet is rich in fiber contents have gut microbes composed of low levels of Firmicutes but high levels of Bacteroidetes (e.g., Prevotella and Xylanibacter) compared with Italian children with a low-fiber diet whose gut flora contains of high levels of Firmicutes and reduced levels of Bacteroidetes [8]. Prevotella and Xylanibacter are associated with SCFAs as they ferment xylan and cellulose through carbohydrate-active enzymes such as xylanase, carboxymethylcellulase and endoglucanase [8]. This indicates that the microbiota of the rural African children has managed to adapt to maximize energy extraction from a diet rich in fiber.

In addition, animal-based diets increase the abundance of Bacteroides, which are associated with protein intake, and decreases the number of Firmicutes such as Roseburia and Ruminococcus bromii [11]. These phenomena reflect the existence of bacterial specificity, for example, that Ruminococcaceae sequences are associated more with the particulate fraction (12.2%) while less with the liquid fraction (3.3%). This relationship was confirmed by fecal samples collected from healthy individuals in a previous study [92]. In contrast, the Gram-negative Bacteroides sequences appeared to partition more with the liquid substrate, suggesting that certain bacteria might represent specialist degraders of certain substrates [58]. Dietary shifts create novel bacterial compositions that achieve maximum nutrition utilization in an adaptable manner that is crucial to the health and development of their host organisms [11,20,21,22,23,93]. However, evidence suggests that long-term diet acts as a strong force on the stability of the composition of the gut microbiota over time [33].

Research has shown that there is also a close coevolution between diet and the digestive tract, a relationship first described in 1972 [13,14,17,94]. Resource quality, abundance and/or composition influences the available trophic niches, which creates strong selection forces for phenotypic divergence through alteration in digestive traits (e.g., gut morphology and physiology). These occur through both phenotypic plasticity and evolutionary mechanisms, and results in increased acquisition of, digestion of, and assimilation of energy and nutrients from dietary items [18,95]. Species adapted to feeding on low-quality food sources are forced into higher levels of food intake to meet nutrition and energy demands [18,94,96,97,98,99]. As a result, much more energy must be expended on feeding activity [100]. The flux of food is accelerated by the high rates of food intake, and thus the time that dietary items remain in the gut is decreased [79]. For example, it was shown that in meadow voles, as energy demand increased, food intake increased by 40%, yet the turnover time to digest food items decreased by 26% [79]. Digested energy increased by 30% in spite of a 4% loss of digestive efficiency and shorter turnover time [79]. In this response, the ratio of energy assimilated to energy ingested, known as the efficiency of gut digestive assimilation, remains stable [36,47].

Without producing extra length in the gut organs, proliferation of existing gut tissue, especially mucosal tissue, only moderately increases nutrient utilization [79]. To digest nutrient-poor diets more efficiently, an extended and voluminous gut has evolved to help herbivores maintain enough retention time [96,97,98,99] when faced with the increased gut passage rates which are required by low-quality food [101]. However, large amounts of energy or nutrition are demanded to balance the investment in increased gut length [102]. Herbivorous animals must thus feed on large quantities of low quality, difficult to digest food, and relatively large guts with voluminous and complicated fermenting chambers (e.g., small intestine) are in turn demanded [15,17,98]. Conversely, smaller guts characterized by simple stomachs and proportionately long small intestines are featured in organisms that tend to feed on diets characterized by smaller quantities of high quality food [13,14,15]. For instance, birds fed a mostly artificial diet with only a small proportion of heather (Calluna vulgaris), their natural food source, saw decreases in the length of both the small and large intestines [14]. The relationship between diet quality and gut size is also evident in primates. Anthropoid primates have relatively larger guts, while humans have relatively smaller guts as they feed mainly on high-quality foods such as underground tubers, meat, and cooking food [15,16,40]. In conclusion, diet quality is an important factor for the evolution of gut traits.

Several studies have also evidenced that gut microbiota plays an important role in determining gut size [20,22,23,103,104]. The ceca of germfree (GF) mice, rats, rabbits, and guinea pigs have been found to be four to seven times larger than those in comparable conventional (CV) animals. In animal species where the cecal sac is not prominent, gut enlargement does not occur with the absence of microbes, but an accumulation of mucus in the lower gut may lead to localized fluidity of the contents and to the presence of bioactive materials similar to those found in the cecal contents of GF rats and mice [22,105].

Intestinal tracts of GF animals usually weigh less than those of CV animals [106,107]. This deficit results mainly from a reduction in lamina propria tissue. In GF rats and mice, the surface area of the small intestine is reduced to approximately two thirds of that of a CV animal [20,107,108]. In pigs and dogs, on the other hand, the surface area of GF and CV animals has been found to be approximately comparable [103,104].

Combined with the previously mentioned evidence that gut microbiota can adapt to changes in diet quality and in turn increase digestion, the idea that the gut microbiota acts as a modulator for gut size gains merit.

## 4. The Expensive-Tissue Hypothesis (ETH): Gut Size and Brain Size

The brain is an energetically expensive organ in the vertebrate body [109] due to its high resource demands, especially in humans [40,53]. In particular, while the adult brain only takes up 2% of total body weight, it consumes about 20% of the body’s energy [110,111]. Compared with the brain of a chimpanzee, the human brain is three times larger and thus much more energy is required. This high level of energetic expenditure is mainly connected with ion pumping, which plays an essential role in maintaining the potentials across the axonal membranes. Additionally, large amounts of energy are applied to the continual synthesis of neurotransmitters [15]. Brain size increases when energy inputs remain at a higher level through enhancing mean diet quality, extra energy intake and/or through maintaining the stability of energy inputs [42,43,112,113,114]. However, in spite of the great differences between the brain size of humans and the chimpanzee, the basal metabolic rates per unit of body weight are very similar between the two species [15,47,115]. There is also no sufficient evidence explaining additional metabolic costs of the large brain in both primate and eutherian mammals [15]. Therefore, a trade-off is needed to solve this emerged situation.

The expensive-tissue hypothesis (ETH), with regard to vertebrate evolution, states that the metabolic requirement of a relatively large brain should be compensated for by a reduction in the size of the gut, which is also an energetically costly organ, and other costly tissues [15,36,37,38,39,40,41,42]. In addition to evidence for the ETH, there have also been studies with results that refuted the theory [43,44,45,46,47,49]. Since its original formulation there have been extensions to the hypothesis, however, the “energy trade-off hypothesis” is one of the proposed extensions of the original ETH [44] that suggests that the cost of increased brain size can be compensated by a costly loss of traits including and other than gut size and digestion, such as body maintenance [45], locomotion [47], development [46], and reproduction [43,49]. Predictions of these hypothesis have been examined by several comparative studies and a negative association between brain and gut size, or negative associations between the brain and other costly tissues, have been found by some studies [36,37,38,40,41,42]. Despite this support of the ETH [36,37,38,40,41,42], other studies have found no such patterns [47,48,50,51].

In 2016, Liao et al. found that, within 30 species of anurans, brain mass was negatively associated with the length of the digestive tract when controlling for phylogenetic relationships and body size [36]. The human brain is relatively larger than those of other mammals, and we also have substantially smaller guts [15,39,42]. An experimental study using guppies (Poecilia reticulata) aimed at searching out divergences in the brain found that increased brain size could result in reduced gut size and fecundity [41].

## 5. Diet, Gut Microbiota, and the Trade-Off between Gut and Brain Size

Numerous studies have addressed the relationship between gut size and brain size [15,36,38,39,41,42,50]. In previous research, some researchers have proposed that social-ecological factors, such as social intelligence, might be capable of explaining the underlying mechanisms of encephalization and brain expansion in primates [116]. However, after taking other issues into consideration, DeCasien et al. concluded that in primates, brain size was affected by diet rather than social factors [117].

Dietary quality affects brain size due to the chemical and energy requirements of the brain [52]. Alterations in diet quality have been proven to be able to affect brain size. For instance, it is likely that a diet consisting of more meat results in increases in brain size with corresponding reductions in gut size [39]. By allowing the existence of a relatively smaller gut, a high-quality diet can succeed in contributing to encephalization and decreasing the metabolic cost of this tissue, thus allowing more energy to be available for fueling a larger brain [15].

Fat plays an essential role in forming the brain and nervous system as well as all other soft tissues. Brain lipids consist of cholesterol and phosphoglycerides with a number of long-chain fatty acids [52]. The long-chain fatty acids needed by the brain can be produced indirectly by the liver whose substrate is provided by gut microbiota [26,29,33]. As has been mention above, gut microbiota can produce SCFAs via fermentation which then compound glucose through gluconeogenesis. Brain glucose metabolism is mainly concentrated on the production of energy from glycolysis and oxidative phosphorylation. Aerobic glycolysis is associated with gene expression controlling earlier phases of brain development [118]. Additionally, the relationship between neurons and astrocytes/oligodendrocytes is reflected by aerobic glycolysis [119,120]. In this relationship, astrocytes take up glutamate in the synaptic cleft in a sodium-dependent process. Lactate is then consumed by the neuron, where it has a profound influence on the allocation of metabolic resources needed in biosynthesis.

Lately, a study has found that brain development is affected by the intestinal microbiota in humanized gnotobiotic mice, suggesting that early neuron and oligodendrocyte development can be affected by growth-associated microbiota [121].

**Figure 3:**
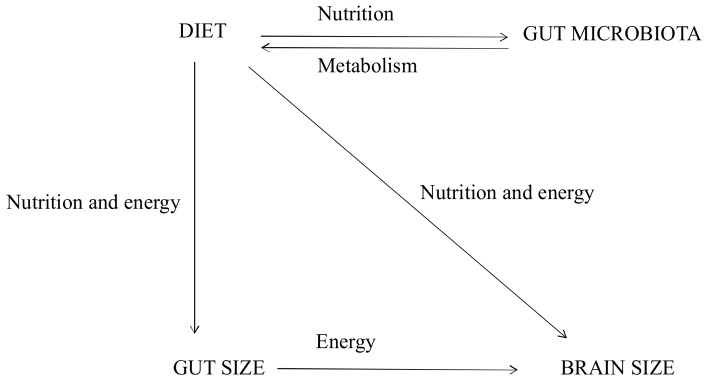
The Relationship Between Gut and Brain Size.

The variation of gut size and the size of other tissues (such as liver and heart) has been proven to be affected by gut microbiota and correlated with dietary intake [22,20,103,104]. Considering this, and assuming the ETH holds merit, it can be concluded that the gut microbiota can mediate the relationship between brain and gut size through both nutrition and energy pathways (Figure 3).

## 6. Future Perspectives

One of the most heavily researched topics in microbiology in recent years is gut microbiota and the underlying mechanisms driving its influence on host metabolism.

Recent research has focused on the effects gut microbiota has on areas of the brain involved in processing emotion [122], neurodegeneration [123,124], behavior [125], and development [121,126] through the gut microbiota-gut-brain axis. The function of gut microbiota along this axis with regard to both energy and chemical substrate production has been investigated. In the future, developing beneficial bacteria to improve brain size and cognitive ability is a potentially attractive research avenue, as well as better exploring and defining the co-interaction of different bacterial species within the microbiota.

However, how gut microbiota influences brain size remains unclear. This is most likely a result of underpowered research that includes participants with diverse and unclear factors that affect the underlying mechanism that controls the relationship between brain size and gut microbiota, which makes the problem even more challenging.

Furthermore, recent evidence indicates that gut microbiota influences brain development, yet there is longer term and more detailed research that is needed to clarify the role played by the gut microbiota in determining brain size and cognitive ability. Such information could be applied to cognitive therapy (e.g., optimizing brain development and function in intellectually challenged children). Achieving this purpose, we require the microbial physiologist’s knowledge, for example, and can utilize the effect of growth phenotype-associated microbiota on growth via the signal pathway of mRNA expression in the brain.

## 7. Conclusions

Numerous studies have revealed remarkable diversity of the microbiota within vertebrate digestive tracts and that there is inter-individual variation in the composition of their respective microbiota communities. The composition of intestine microbiota is determined by dietary intake and can change the size of organs, especially gut size. This is important to consider in combination with the massive energy demands of encephalization and brain enlargement. These costs of increased brain size can be met via increasing overall energy intake or by changing relative energy allocations. Therefore, gut microbiota may affect brain size via increasing energy intake and reducing gut size, which would support the ETH. However, it remains challenging to find evidence to support the conclusion that brain size is modified by gut microbiota. Achieving this feat will depend on the development of advanced measurement technology and the long-term improvement of artificial cultures of germ-free animals.

## Abbreviations

ETH	the Expensive-Tissue Hypothesis
SCFAs	short-chain fatty acids
FFAR2	free fatty acid receptor 2
FFAR3	free fatty acid receptor 3
PYY	peptide YY
NPY	neuropeptide Y
GLP1	glucagon-like peptide 1
POMC	anorexigenic pro-opiomelanocortin
HDAC	histone deacetylase
CK2	casein kinase II
Sp1	specificity proteins 1
Sp3	specificity proteins 3
GF	germ free
CV	conventional
